# Comparison of the 6th and 7th editions of the AJCC/UICC TNM staging system for gastric cancer focusing on the “N” parameter-related survival: the monoinstitutional NodUs Italian study

**DOI:** 10.1186/s12957-015-0633-3

**Published:** 2015-07-16

**Authors:** Luigi Marano, Virginia Boccardi, Bartolomeo Braccio, Giuseppe Esposito, Michele Grassia, Marianna Petrillo, Modestino Pezzella, Raffaele Porfidia, Gianmarco Reda, Angela Romano, Michele Schettino, Angelo Cosenza, Giuseppe Izzo, Natale Di Martino

**Affiliations:** 8th General and Gastrointestinal Surgery, Department of Internal Medicine, Surgical, Neurological Metabolic Disease and Geriatric Medicine, Second University of Naples, Piazza Miraglia 2, 80138 Naples, Italy

**Keywords:** Gastric cancer, Staging system, Lymph node status, 7th TNM, 6th TNM

## Abstract

**Background:**

A large number of Asian population studies examined the difference between the 6th and the 7th tumor, node, metastasis (TNM) while it is still poorly validated among Caucasian populations. This is a retrospective study aimed at investigating the efficacy of the 7th edition American Joint Committee on Cancer (AJCC)/Union for International Cancer Control (UICC) staging system for gastric cancer focusing on the “N” parameter-related survival for prognostic assessment in gastric cancer patients of a single Western high-volume institution.

**Methods:**

From January 2002 to December 2009, the data of 274 patients with gastric cancer who underwent gastric surgery at the 8th General and Gastrointestinal Surgical Centre of the Second University of Naples were analyzed retrospectively. We collected data for patient demographics, tumor characteristics, surgical characteristics, and TNM stage. Particularly, the nodal status, with the number of dissected nodes and metastatic nodes, was reviewed from the pathology records. The same patient dataset was used to stage patients according to both the 6th and 7th edition criteria.

**Results:**

Age at surgery, tumor location, histological grade, Lauren’s classification subtypes, and 6th and 7th AJCC/UICC N categories were found to have statistically significant associations with overall survival on univariate analysis. In the 6th edition staging system, the Kaplan–Meier plot did not show significant overlapped survival curves: significant differences were found between N0 and N1, *P* < .001; N1 and N2, *P* = .04; and N2 and N3, *P* < .001. On the contrary, in the 7th edition, among all five substages, there were similar survival curves between N categories 2 and 3a (*P* = .98) with a statistically significant discriminatory ability only between N1 versus N3b and N2 versus N3b (*P* = .02 and .04, respectively).

**Conclusions:**

Based on analysis, we found that several clinicopathological variables, especially histological grade and Lauren’s classification, were significant prognostic factors in our database. The 6th and 7th AJCC/UICC N classifications represent significantly independent prognostic factors, and the 6th AJCC/UICC N classification seems to be superior to the 7th AJCC/UICC N classification in terms of uniformity, differentiation, and monotonicity of gradients.

## Background

Gastric cancer is one of the most common malignant tumors in the world and the second leading cause of cancer-related death worldwide [1–5]. Although considerable progress has been made in the early gastric cancer diagnosis, so far, the prognosis of this tumor remains poor [6, 7].

Accurate categorization of the tumor stage, including the invasive depth and lymph node status, is crucial for prognostic assessment and decision-making of the stage-specific therapeutic strategy [8]. The American Joint Committee on Cancer (AJCC)/Union for International Cancer Control (UICC) tumor, node, metastasis (TNM) staging system has been used widely for clinical practice and research in determining tumor stage for gastric cancers, representing the most important independent prognostic factor [9]. Several versions of this classification system have been used over the past 30 years [10, 11], and in 2010, the 7th edition of the AJCC/UICC gastric cancer staging manual was introduced, resulting in several changes from the 6th edition, particularly as regards the N categories [12]. According to the new TNM edition, N categories were so redefined in order to improve their reproducibility and prognostic validity: the previous N1 category (1–6 involved regional lymph nodes) is amended into a new N1 category (1–2 involved regional lymph nodes) and N2 category (3–6 involved regional lymph nodes), whereas the previous category N2 (7–15 involved regional lymph nodes) and the N3 category (>15 involved regional lymph nodes) are combined into the new N3 categories (N3a: 7–15 involved regional lymph nodes, and N3b: ≥16 involved regional lymph nodes) [2, 11]. In the medical literature, there are a large number of Asian population studies that examine the difference between the 6th and the 7th TNM [2, 11, 13–15]; however, gastric cancer in Western countries represents a different disease considering the pattern of presentation and pathophysiology [16–18]. Indeed, studies aimed at validating the new staging criteria focusing on the N category in the Italian population are poor [19]. Thus, the prognostic capability of this new classification in the Western remains ambiguous. In light of such evidences, we performed a retrospective study so-called NodUs study (Nodal statUs) to evaluate the efficacy and validity of the 7th edition AJCC/UICC “N” category for prognostic assessment and to compare the 6th and 7th editions of the AJCC/UICC “N” staging system focusing on the “N” parameter in a cohort of patients who underwent surgical therapy for gastric cancer from a single Western high-volume institution, providing reference for revision of a future edition of the AJCC/UICC for gastric cancer staging.

## Methods

From January 2002 to December 2009, the data of 329 patients with gastric adenocarcinoma (ICD-O code 8140/3 according to the World Health Organization classification of tumors of the digestive system) [20] confirmed by histopathology who underwent gastric surgery at the 8th General and Gastrointestinal Surgical Centre of the Second University of Naples were analyzed retrospectively. Exclusion criteria were previous history of surgery for gastric cancer, gastric stump cancer, metastatic disease, non-curative resection (R1 or R2 resection), lymphadenectomy different from D2, inadequate lymph node dissection (<15 lymph nodes retrieved), preoperative radiotherapy, and/or chemotherapy. More specifically, in D1 dissections, only the perigastric nodes directly attached along the lesser curvature and greater curvatures of the stomach are removed (stations 1–6: right and left pericardial, lesser curvature, greater curvature, supra, and infrapyloric). D2 dissections add the removal of nodes along the left gastric artery (station 7), common hepatic artery (antero-superior group, station 8a), celiac trunk (station 9), splenic hilum and splenic artery (station 10 and 11), and hepatoduodenal ligament (along the proper hepatic artery, station 12a) [21].

After applying the exclusion criteria, a total of 274 patients were enrolled in this study.

All patients underwent standardized total or partial gastrectomy (depending on the distance between the cardia and the tumor) by experienced surgeons, with spleen-preserving modified radical D2 lymphadenectomy according to the Japanese Classification of Gastric Carcinoma [21]. However, splenectomy was necessarily performed in a total of 19 patients (6.9 %) to ensure complete dissection of the splenic hilar lymph nodes in very difficult dissection cases. The surgical procedure of reconstruction was chosen according to the surgeon’s preference. We collected data for patient demographics, tumor characteristics, surgical characteristics, and TNM stage. Particularly, the nodal status, with the number of dissected and metastatic nodes, was reviewed from the pathology records. The same patient dataset was used to stage patients according to both the 6th and 7th edition criteria.

Surviving patients were followed at regular intervals at the outpatient clinic until 5 years after surgery. Outpatient clinic visits included history taking and physical examination. No routine imaging was performed. Overall survival, used as a prognostic parameter, was defined as the time between the date of operation and the date of death. Surviving patients were censored on the day of last follow-up. The last follow-up checkpoint was July 2014.

### Ethics, consent, and permissions

The study was approved by the ethics committee of the Second University of Naples and conducted according to the ethical standards of the Helsinki Declaration. All patients gave informed consent to participate in this study.

### Consent to publish

Consents to publish have been obtained from the participants (or legal parent when appropriate) to report individual patient data.

### Statistical analysis

The observed data were normally distributed. Overall survival (OS) was calculated using the Kaplan–Meier method, and the log-rank test was employed to determine the significance.

Factors that were deemed of potential importance on univariate analysis, considering a *P* value of less than .05 as a statistically significant result, were included in multivariate analyses. Multivariate analysis was performed by the Cox proportional hazard model using the forward logistic regression stepwise procedure for variable selection. To measure homogeneity of the direct comparison of the two different edition stage systems, the likelihood ratio *χ*^2^ test related to the Cox regression model was used. The discriminatory ability and monotonicity of gradient assessments were measured with the linear trend *χ*^2^ test of survival curves according to the N classification of the 6th and 7th editions. The Akaike information criterion (AIC) was applied into the Cox proportional hazard regression model to correct for the potential bias in comparing prognostic systems with different numbers of stages. AIC was defined as follows: AIC = −2 log maximum likelihood + 2 × (the number of parameters in the model). A smaller AIC value indicated a better model for predicting outcome [22, 23]. Hazard ratios (HR) and 95 % confidence intervals (95 % CI) were generated. A statistical data analysis was performed using SPSS 20.0 software (SPSS Inc., Chicago, IL), and a *P* value of less than .05 was considered to be statistically significant.

## Results

**Fig. 1:**
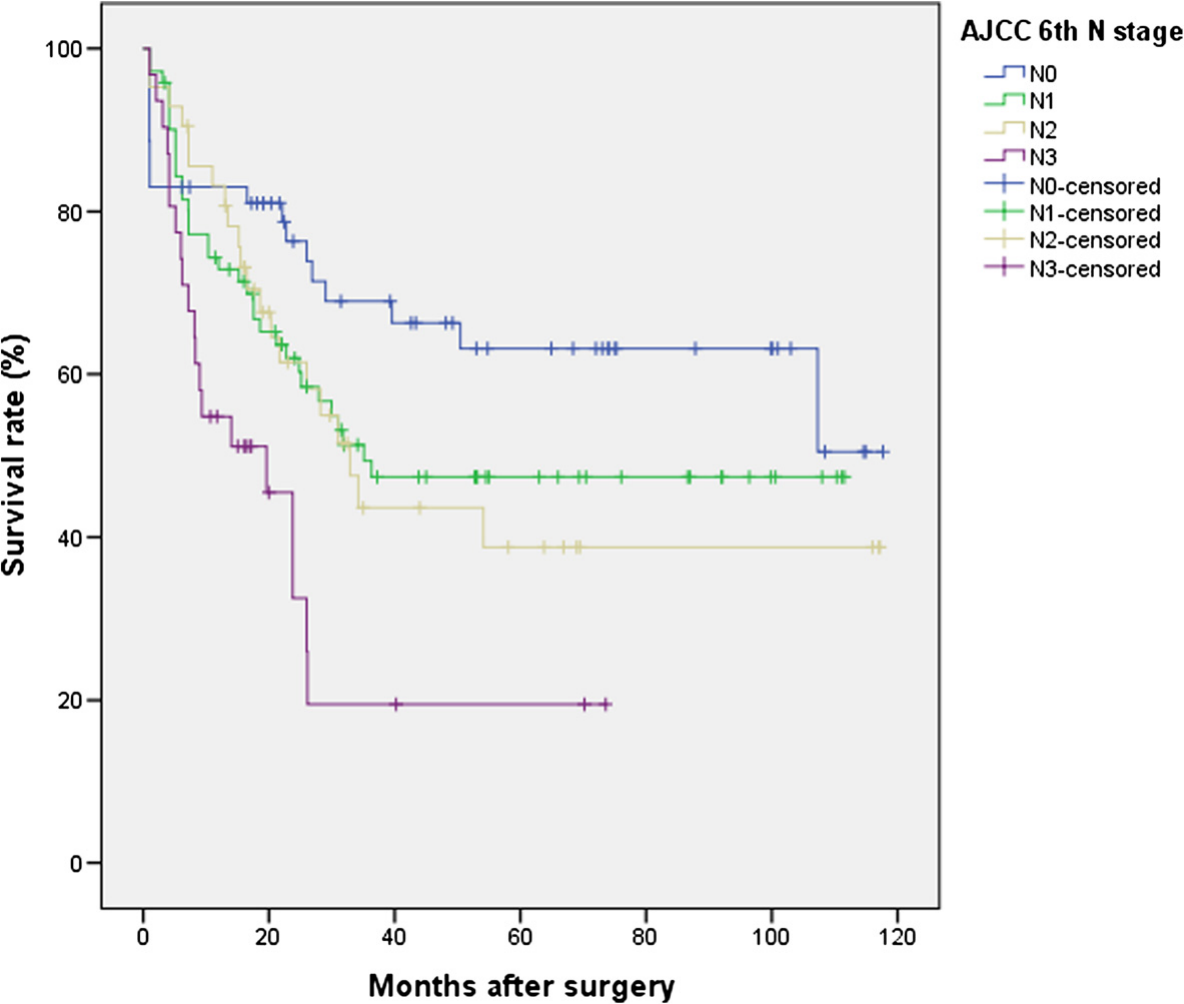
Survival curves for 274 patients according to the 6th AJCC N classification

**Fig. 2:**
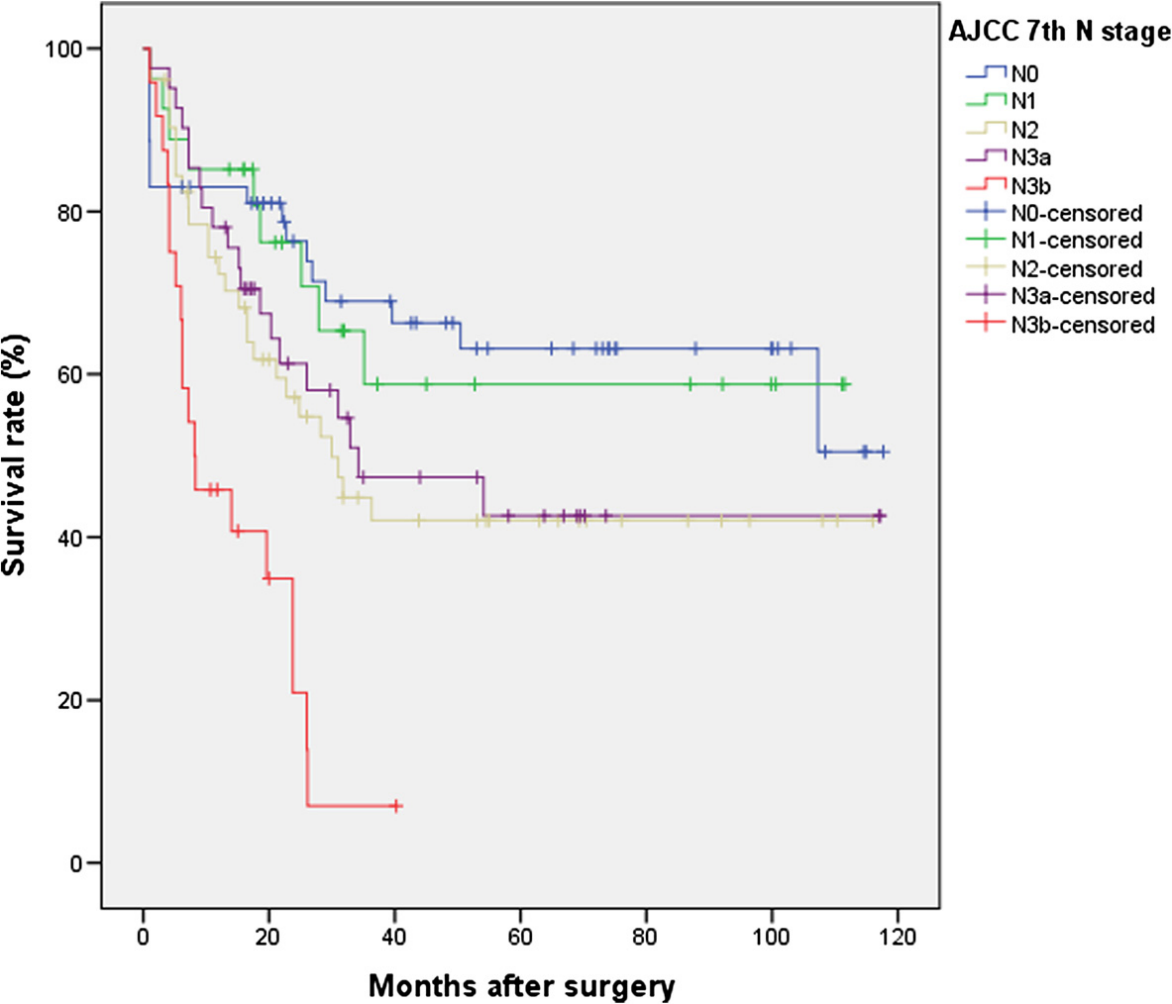
Survival curves for 274 patients according to the 7th AJCC N classification

A consecutive series of 329 patients with diagnosis of gastric adenocarcinoma was analyzed retrospectively. In total, 20 patients were excluded because they had received neoadjuvant chemotherapy, 8 patients were excluded because of a non-curative (R1) resection, and 17 patients were excluded because of inadequate lymph node dissection and lymphadenectomy different from D2 (D1 lymphadenectomy). Ten patients had a metastatic disease and were also excluded from the current analysis. This resulted in a final study population of 274 patients. Mean follow-up was 53 months (median 39 months). The overall 5-year survival rate was 52.8 %. Age at surgery, tumor location, histological grade, Lauren’s classification subtypes, and 6th and 7th AJCC/UICC N parameters were found to have statistically significant associations with overall survival on univariate analysis. Patient characteristics and the effect of clinical features on survival are summarized in Table 1. No lymph node metastasis was found in 66 patients (23.9 %). The number of metastatic nodes was 1–6 (N1) in 103 patients (37.6 %), 7–15 (N2) in 61 patients (22.2 %), and more than 15 (N3) in 44 patients (16.2 %) according to the TNM 6th criteria. The number of metastatic nodes was 1–2 (N1) in 44 patients (16.2 %), 3–6 (N2) in 71 patients (26.1 %), 7–15 (N3a) in 60 patients (21.8 %), and more than 15 (N3b) in 33 patients (12 %) according to the new TNM 7th criteria. The total number of dissected lymph nodes was 7267, with an average of 26.5 ± 14.8 (mean ± SD) dissected nodes per case (median 24.0, range 0–87). The mean number of metastatic nodes was 6.5 ± 8.2 (median 4, range 0–55) in the overall series and 8.5 ± 8.4 (median 6, range 1–55) in lymph node-positive patients (data not shown). Figure 1 shows the 5-year survival rates for patients with N0 (66.0 %), N1 (52.1 %), N2 (50.0 %), and N3 (31.0 %) disease (*P* < .001) according to the AJCC/UICC 6th edition system. Also shown in Fig. 2 are the 5-year survival rates according to the AJCC/UICC 7th edition: N0 (66.0 %), N1 (66.7 %), N2 (48.1 %), N3a (51.2 %), and N3b (20.8 %) (*P* < .001). Indeed, in the 6th edition staging system, the Kaplan–Meier plot did not show significant overlapped survival curves: significant differences were found between N0 and N1, *P* < .001; N1 and N2, *P* = .04; and N2 and N3, *P* < .001. On the contrary, in the 7th edition, among all five substages, there were similar survival curves between N categories 2 and 3a (*P* = .98) with statistically significant discriminatory ability only between N1 versus N3b and N2 versus N3b (*P* < .001 and .04, respectively).

**Table 1:** Univariate survival analysis of clinicopathological variables in 274 gastric cancer patients

	*N* (%)	5-year survival rate (%)	Log-rank *χ* ^2^ value	*P* value
Gender			1.987	0.159
Female	95 (34.7)	50.6		
Male	179 (65.3)	42.7		
Age at surgery (years)			3.247	0.047
≤40	14 (5.1)	80.0		
41–60	90 (32.8)	41.9		
>61	170 (62.1)	44.5		
Tumor location			4.786	0.025
Upper	77 (28.1)	43.1		
Upper-middle	34 (12.4)	38.7		
Middle	72 (26.3)	42.9		
Middle-lower	29 (10.6)	41.7		
Lower	62 (22.6)	56.4		
Histological grade			6.788	0.009
Well + moderately differentiated	129 (47.1)	51.7		
Poorly differentiated + signet ring cell	145 (52.9)	38.7		
Lauren’s classification			9.772	0.002
Intestinal type	104 (38.0)	54.7		
Diffuse type	170 (62.0)	39.7		
Type of gastrectomy			0.656	0.199
Subtotal	40 (14.6)	47.1		
Total	234 (85.4)	45.2		
The 6th N stage (AJCC)			17.013	0.002
N0	66 (23.9)	66.0		
N1	103 (37.6)	52.1		
N2	61 (22.2)	50.0		
N3	44 (16.2)	31.0		
The 7th N stage (AJCC)			29.483	0.0003
N0	66 (23.9)	66.0		
N1	44 (16.2)	66.7		
N2	71 (26.1)	48.1		
N3a	60 (21.8)	51.2		
N3b	33 (12.0)	20.8		

*AJCC* American Joint Committee on Cancer, *N* node

All six variables that resulted in statistical significance associated with overall survival on univariate analysis were included in a multivariate Cox proportional hazard model with forward logistic regression stepwise procedure to adjust for the effects of covariates. Therefore, two separate multivariate models, one with the 6th and the other with the 7th AJCC/UICC N classification, were run to avoid collinearity problems. In these models, we demonstrated that histological grade, the 6th AJCC N parameters, and the 7th AJCC/UICC N parameters were independent factors of prognosis of gastric cancer (Table 2). After that, a Cox regression model including both the 6th and 7th edition staging systems focusing on the “N” parameter showed that the 7th edition no longer significantly predicted survival, whereas the 6th edition remained a significant stratifier of prognosis (data not shown). Finally, the performance of the 6th and 7th edition N classification systems was quantified by the likelihood ratio chi-square, linear trend *χ*^2^ test, and AIC. Predictive ability was best for the 6th AJCC/UICC N classification system (highest likelihood ratio *χ*^2^) as well as the discriminatory ability and monotonicity of gradients (higher linear trend *χ*^2^ score). Furthermore, the AIC value was smaller for the 6th edition compared to the 7th edition staging system, indicating that it has a better prognostic stratification (Table 2).

**Table 2:** Two multivariate analysis models of overall survival in gastric cancer patients

Variables	Hazard ratio	95 % CI	*P* value
(a) 6th AJCC N staging system (−2 log likelihood: 1548.021; linear trend *χ* ^2^: 1501.231; AIC value: 2591.3)			
Age at surgery	1.025	0.748–1.598	0.652
Tumor location	0.984	0.548–1.746	0.985
Histological grade	0.745	0.624–1.348	0.015
Lauren’s classification	0.864	0.521–1.681	0.312
The 6th N stage (AJCC)	1.256	0.894–1.612	0.007
(b) 7th AJCC N staging system (−2 log likelihood: 1541.013; linear trend *χ* ^2^: 1498.564; AIC value: 2601.4)			
Age at surgery	1.101	0.809–1.494	0.884
Tumor location	1.003	0.756–1.319	0.731
Histological grade	0.639	0.517–1.214	0.021
Lauren’s classification	0.931	0.611–1.306	0.424
The 7th N stage (AJCC)	1.481	1.112–2.031	0.011

*95 % CI* 95 % confidence interval, *AJCC* American Joint Committee on Cancer, *AIC* Akaike information criterion, *N* node

## Discussion

The major finding of our investigation on 274 gastric cancer Italian patients who underwent primary surgical resection is that the 6th and 7th AJCC/UICC N classifications represent significantly independent prognostic factors, and the 6th AJCC/UICC N classification seems to be superior to the 7th AJCC/UICC N classification in terms of uniformity, differentiation, and monotonicity of gradients. Nowadays, the prognosis for gastric cancer patients remains poor, and the TNM stage system represents a prognostic factor that can effectively provide the means for appropriate treatment and predict the prognosis [23, 24]. Especially the extent of lymph node metastasis is proved to be the most important independent prognostic factor [25, 26]. The introduction of the new 7th AJCC/UICC TNM edition has brought several changes for gastric cancer classification, and 4 years later, there are still some debates about its prognostic power. Furthermore, in the literature are reported scientific contributions with controversial results about comparison between the 6th and the 7th AJCC/UICC N classification systems. Wang et al. [1] reported a better prognostic stratification of the latest TNM edition in 1503 gastric cancer patients who underwent primary surgical resection with an average per case of dissected lymph nodes less than 15, considering avoidable N3 substratification in N3a and N3b. Similarly, Deng et al. [27], Chae et al. [28], and Fang et al. [15] showed a more detailed classification of different prognostic groups with a high homogeneity rate in each TNM stage in R0-selected patients with more than 16 retrieved lymph nodes per case [29], assessing prognostic superiority for the 7th than for the 6th AJCC/UICC N classification system. From the Western point of view, McGhan et al. [25] evidenced a better survival discrimination and risk stratification of the 7th AJCC/UICC staging criteria in a retrospective review of 13,547 American gastric cancer patients. In order to evaluate the efficacy and validity of the 7th edition AJCC/UICC “N” classification system for prognostic assessment and to compare the 6th and 7th editions of the AJCC/UICC “N” classification system, we analyzed 274 patients who underwent curative surgery by experienced surgeons in this “NodUs” retrospective study.

Our data analysis, different from Asian surgeons’ results, showed that survivals according to the 6th AJCC/UICC N classification were more equally distributed than survivals according to the new AJCC/UICC classification. Moreover, in univariate analysis, significant prognostic factors were the age at surgery, tumor location, histological grade, Lauren’s classification, the 6th AJCC/UICC N category, and the 7th AJCC/UICC N category. However, the two multivariate analysis models showed that histological grade, the 6th AJCC/UICC N classification, and the 7th AJCC/UICC N classification were independent factors of prognosis of gastric cancer. Furthermore, when a Cox regression model including both the 6th and 7th edition staging systems focusing on the “N” parameter was run, only the 6th AJCC/UICC N classification remained a significant stratifier of prognosis with high prediction as well as the discriminatory ability and monotonicity of gradients. Similarly, some Eastern surgeons did not report the 7th AJCC/UICC N classification as having a more effective prognostic power compared to the 6th edition [2, 4, 30]. Likewise, Rausei et al. [31] in a retrospective unique Italian “real-world” comparative study on 224 non-metastatic gastric cancer patients who underwent surgery with curative intent and limited lymphadenectomy (D1 lymphadenectomy) did not find any prognostic superiority of the 7th AJCC/UICC TNM edition with respect to the N parameter in comparison to the 6th edition. As stated by Bickenbach et al. [16] and McGhan et al. [25], gastric cancer in Western countries represents a different disease as regards the pattern of presentation and pathophysiology making the classification according to the new 7th AJCC/UICC N classification system not better than the old 6th TNM for the prognostic stratification of staging in gastric cancer. Further nationwide studies with larger numbers of patients are necessary to evaluate the validity and effectiveness of the new classification from various angles.

## Conclusions

Although our sample population comes from a single institution experience and is relatively small compared with the worldwide gastric cancer collaboration database, the surgical procedures, pathologic examinations, and patient follow-up were highly uniform throughout the entire study period. Moreover, it represents an original retrospective study on the Italian population. In our analysis, we found that several clinicopathological variables, especially histological grade and Lauren’s classification, were significant prognostic factors in our database worthy of further research. Overall, the 6th and 7th AJCC/UICC N classifications represent significantly independent prognostic factors, and the 6th AJCC/UICC N classification seems to be superior to the 7th AJCC/UICC N classification in terms of homogeneity, discriminatory ability, and monotonicity of gradients, providing reference for revision of a future edition of the AJCC/UICC for gastric cancer staging.

## Abbreviation

AIC: Akaike information criterion
AJCC: American Joint Committee on Cancer
CI: confidence interval
HR: hazard ratio
OS: overall survival
TNM: tumor, node, metastasis
